# Association between visual function and the integrity of residual ellipsoid zone in resolved central serous chorioretinopathy

**DOI:** 10.1038/s41598-019-48825-7

**Published:** 2019-08-27

**Authors:** Asahi Fujita, Yurika Aoyama, Saori Tsuneyoshi, Aya Sugiura, Keiko Azuma, Kimiko Asano-Shimizu, Hirotsugu Soga, Yohei Hashimoto, Ryo Asaoka, Tatsuya Inoue, Ryo Obata

**Affiliations:** Department of Ophthalmology, The University of Tokyo Hospital, Tokyo, Japan

**Keywords:** Eye diseases, Outcomes research, Eye manifestations

## Abstract

Central serous chorioretinopathy (CSC) usually resolves spontaneously; however, in some patients, it can be chronic and visual impairment remains even after resolution of the serous retinal detachment. The impaired photoreceptor cells often present with disrupted ellipsoid zone (EZ) on optical coherence tomography (OCT). In this study, the integrity of EZ was quantified by calculating the index of residual EZ, identified on binarized OCT images from 25 eyes of 23 patients with resolved CSC. To estimate residual EZ, integrity of residual EZ with the central horizontal line on the fovea (rEZc) and average integrity of residual EZ within 3 × 3-mm macular area (rEZave) were investigated. The interrater reliability of the method was assessed using the intraclass correlation coefficient (ICC). The relationship between LogMAR VA and age, central retinal thickness, central choroidal thickness, rEZc, and rEZave were evaluated using the linear mixed model. Retinal sensitivity was measured with the MP-3 microperimeter and similar analyses were iterated for mean retinal sensitivity (MS). ICC values were 0.938 with rEZc and 0.979 with rEZave. rEZc was significantly related to LogMAR VA (p = 0.039). rEZave was significantly related to MS (p < 0.001). These results suggested potential usefulness of residual EZ to predict visual function in resolved CSC.

## Introduction

Central serous chorioretinopathy (CSC) is an eye disease with serous retinal detachment (SRD) induced by leakage through the retinal pigment epithelium (RPE) into the subretinal space, causing decreased visual acuity (VA)^1–3^. Although CSC usually resolves spontaneously, or after either retinal photocoagulation or photodynamic therapy, some symptoms may persist after the resolution of SRD, such as decreased retinal sensitivity (RS) and VA and metamorphopsia^4–7^.

**Figure 1:**
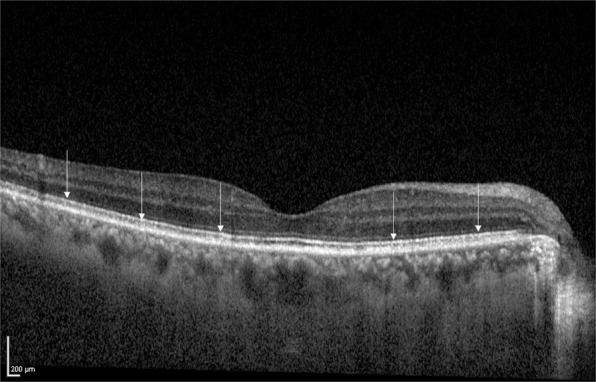
EZ on an OCT image obtained by Spectralis OCT. White arrows indicate EZ on OCT image. EZ: ellipsoid zone, OCT: optical coherence tomography.

The ellipsoid zone (EZ), formerly known as the inner/outer segment of photoreceptors (IS/OS), refers to the second hyper-reflective band on an optical coherence tomography (OCT) image (Fig. 1)^8,9^. The development of OCT imaging technology has enabled us to precisely identify the EZ line^10–13^. Previous reports have indicated that the integrity of EZ was associated with visual outcomes in various retinal diseases^14–18^, such as cone dystrophy^19^, achromatopia^20^, and age-related macular degeneration (AMD)^8,9^. Recent studies revealed that the association of the integrity of EZ with VA and RS in resolved CSC is notable^5,21,22^. However, in these reports, the integrity of EZ was assessed only qualitatively, but not quantitatively.

In the current study, to quantify the residual EZ in patients with resolved CSC, we applied the binarization technique to the OCT images. As a result, the integrity of EZ was successfully measured quantitatively in all eyes. Consequently, quantitative assessment of EZ with the proposed method had a high interrater reliability and a significant structure-function relationship. The quantitative method using binarization technique would be useful to predict visual function in macular diseases.

## Results

### Patient demographics

The demographic data is given in Table 1. The mean age of the patients was 56.9 years (standard deviation, SD: 11.8, range: 33–80). The mean retinal sensitivity (MS) was 25.2 ± 3.30 [18.4–29.6] dB. Central retinal thickness (CRT) and central choroidal thickness (CChT) were 194 ± 49.5 [112–298] μm, 330 ± 95.5 [169–513] μm, respectively. rEZc was 0.785 ± 0.230 [0.0846–1]. rEZave was 0.759 ± 0.239 [0.240–0.992]. The numbers of eyes showing rEZc of <0.4, 0.4 to 0.7, and >0.7 were 1, 8, 16 eyes, respectively. Univariate analysis showed a significant correlation between LogMAR VA and MS (p < 0.01, linear mixed model).

**Table 1:** Demographic data of the patients.

Variables	Mean ± SD [range]
Gender (Male: Female)	15: 8
Age (years)	56.9 ± 11.8 [33–80]
LogMAR VA	0.0748 ± 0.166 [−0.0792–0.523]
MS (dB)	25.2 ± 3.30 [18.4–29.6]
CRT (μm)	194 ± 49.5 [112–298]
CChT (μm)	330 ± 95.5 [169–513]
rEZc	0.785 ± 0.230 [0.0846–1]
rEZave	0.759 ± 0.239 [0.240–0.992]

LogMAR, logarithm of the minimum angle of resolution; VA, visual acuity; MS, mean sensitivity; CRT, central retinal thickness; CChT, central choroidal thickness; rEZc, the rEZ index of the central horizontal line scan across the fovea; rEZave, the average rEZ index of 13 line scans within 3 × 3-mm area in each eye.

### The interrater agreement

**Figure 2:**
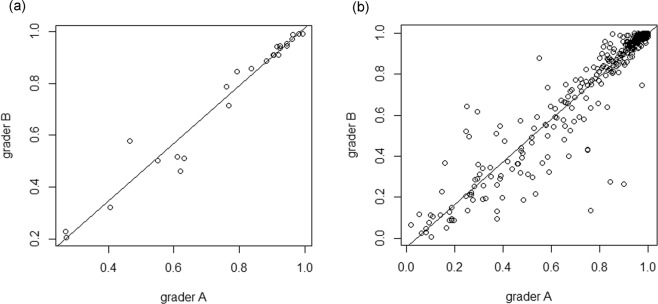
Scatter plots of the integrity of EZ quantified by two graders. Comparison of the measurement of the rEZ index between 2 graders are shown. (**a**) rEZc, the ICC value was equal to 0.938 (95%CI: 0.916–0.945, p < 0.001). (**b**) rEZave, the ICC was 0.979 (95% CI: 0.936–0.987, p < 0.001). EZ: ellipsoid zone, rEZ: residual ellipsoid zone, rEZc: the rEZ index of the central horizontal line scan across the fovea, ICC: intraclass correlation coefficient, CI: confidence interval, rEZave: the average of rEZ indices of 13 line scans within 3 × 3-mm area.

The intraclass correlation coefficient (ICC) of residual EZ with the central horizontal line on the fovea (rEZc) was 0.938 [95% confidence interval (CI): 0.916–0.945, p < 0.001) (Fig. 2(a))] whereas that of average integrity of residual EZ within 3 × 3-mm macular area (rEZave) was 0.979 [(95% CI: 0.936–0.987, p < 0.001) (Fig. 2(b))].

### Relationship between OCT parameters and visual function

With univariate analysis, rEZc, rEZave, and CRT were significantly correlated to LogMAR VA, (linear mixed model, p = 0.0125, p = 0.0322, and p = 0.0180, respectively) whereas CChT was not significantly correlated to LogMAR VA (Table 2). As a result of Akaike information criterion, second order, (AICc) model selection, rEZc and CRT were selected as the best explanatory variables for LogMAR VA (AICc = −17.6, Table 2);

**Table 2:** Univariate and multivariate analyses between LogMAR VA and OCT parameters.

Variables	Univariate analysis			Multivariate analysis		
	Estimate	Standard error	p-value	Estimate	Standard error	p-value
Age	1.31e-03	2.94e-0.3	0.660	N.S.	N.S.	N.S.
rEZc	−0.357	0.132	0.0125	−0.284	0.129	0.0386
rEZave	−0.300	0.132	0.0322	N.S.	N.S.	N.S.
CRT	−0.00163	0.000638	0.0180	−0.00125	0.000616	0.0555
CChT	−0.0000737	0.000364	0.841	N.S.	N.S.	N.S.

LogMAR, logarithm of the minimum angle of resolution; VA, visual acuity; OCT, optical coherence tomography; rEZc, the rEZ index of the central horizontal line scan across the fovea; rEZave, the average rEZ index of 13 line scans within 3 × 3-mm area in each eye; CRT, central retinal thickness; CChT, central choroidal thickness; N.S., not selected.

LogMAR VA = 0.535–0.284 x rEZc (Standard error (SE) = 0.129, p = 0.0386) − 0.00125 × CRT (SE = 0.000616, p = 0.0555).

Excluding the variable of rEZc resulted in a significant decrease of the log-likelihood compared to the optimal model (p = 0.0258, ANOVA).

Among rEZave, rEZ1mm, CRT and CCT, AICc model selection resulted in selecting rEZ1mm as the best explanatory variable for LogMAR VA (AICc = −23.4).

**Figure 3:**
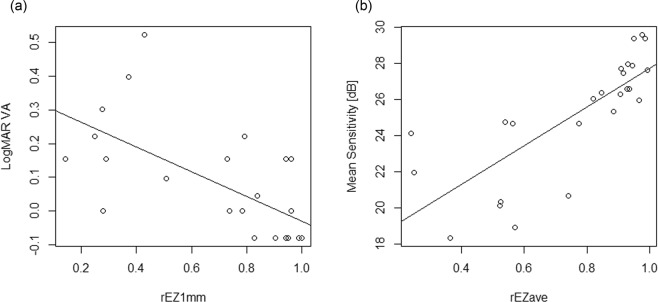
Scatter plots showing the relationship between the integrity of EZ and visual function. (**a**) The relationship between rEZ1mm and LogMAR VA. (LogMAR VA = 0.337–0.366 x rEZ1mm, SE = 0.0891, p < 0.001, linear regression). (**b**) The relationship between rEZave and MS. (MS (dB) = 17.0 + 10.7 x rEZave, SE = 1.83, p < 0.001, linear regression). EZ: ellipsoid zone, rEZ1mm: the average of rEZ indices of 5 line scans within 1 × 1-mm area of the fovea, LogMAR: logarithm of the minimum angle of resolution, VA: visual acuity, rEZave: the average of rEZ indices of 13 line scans within 3 × 3-mm area, SE: standard error, MS: mean sensitivity.

LogMAR VA = 0.337–0.366 × rEZ1mm (SE = 0.0891, p < 0.001, Fig. 3(a)).

Univariate analysis also indicated that rEZc, rEZave and macular volume (MV) were significantly correlated to MS (linear mixed model, p < 0.01, respectively), whereas CChT was not significantly correlated with MS (Table 3). As a result of AICc model selection, age and rEZave were selected as explanatory variables for MS (AICc = 116, Table 3);

**Table 3:** Univariate and multivariate analyses between MS and OCT parameters.

Variables	Univariate analysis			Multivariate analysis		
	Estimate	Standard error	p-value	Estimate	Standard error	p-value
Age	−0.0727	0.0600	0.240	−0.0721	0.0347	0.0497
rEZc	10.7	1.99	<0.001	N.S.	N.S.	N.S.
rEZave	10.7	1.83	<0.001	10.7	1.72	<0.001
MV	8.61	2.24	0.004	N.S.	N.S.	N.S.
CChT	−0.00338	0.00704	0.635	N.S.	N.S.	N.S.

MS, mean sensitivity; OCT, optical coherence tomography; rEZc, the rEZ index of the central horizontal line scan across the fovea; rEZave, the average of rEZ indices of 13 line scans within 3 × 3-mm area in each eye; MV, macular volume; CChT, central choroidal thickness; N.S., not selected.

MS (dB) = 21.2–0.0721 × age (SE = 0.0347, p = 0.0497) + 10.7 × rEZave (SE = 1.71, p < 0.001).

Excluding the variable of rEZave resulted in a significant decrease of the log-likelihood compared to the optimal model (p < 0.01, ANOVA, Fig. 3(b)).

## Discussion

In this study, we quantified the integrity of residual EZ in patients with resolved CSC and analysed the relationship between the quantified EZ integrity and visual function. Based on AICc model selection, rEZc and CRT were selected as the best explanatory variables for LogMAR VA, and age and rEZave were designated for MS.

The usefulness of the assessment of residual EZ has been increasingly reported in recent reports especially of eyes with retinitis pigmentosa (RP). Hood and associates suggested that the point where EZ disappears provides a structural marker for visual field (VF)^23^. Birch *et al*. measured the width of the EZ and suggested its potential usefulness as surrogate for the deterioration of VF^24^. Smith *et al*. reported that EZ width and RS are correlated^25^. Later, Hariri *et al*. obtained en-face OCT images and assessed the residual EZ area, expecting that their method could be used for monitoring the EZ changes of patients with advanced RP^26^. In RP, the peripheral retina is predominantly and diffusely involved such that the damage in the visual function starts at the periphery gradually expanding to the central area with progression of the disease. This implies that the assessment of the residual EZ area is an appropriate approach when monitoring the progression of RP^27^. In contrast, CSC usually occurs in the macular area with the decrease of cone density. A previous report has suggested that the disappearance of the normal cone mosaic was coincident with the disruption of EZ^28^. Therefore, the integrity of EZ in the macula, not the EZ area, would be more predictive of visual function in resolved CSC. In corroboration, our results suggested that the integrity of residual EZ was significantly related to both LogMAR VA and RS measured with the MP-3 microperimeter.

The correlation between the integrity of EZ and visual function has been investigated in resolved CSC in previous studies. Ojima *et al*. reported that areas with defective EZ tend to have decreased RS measured using MP-1 microperimeter^29^. Matsumoto and associates reported that resolved CSC eyes with worse VA had discontinuity of EZ more frequently than those with better VA^21^. Chung *et al*. divided resolved CSC eyes into two groups, those with intact EZ and those with disruptive EZ. The RS and retinal thickness of the disruptive EZ group were significantly lower than those of the intact EZ group^5^. Hence, the integrity of EZ appears to be a critical factor for visual function in resolved CSC. However, in all these reports, the integrity of EZ was assessed only qualitatively, not quantitatively. In these reports, the integrity of EZ was determined as continuous/discontinuous or intact/damaged; but in some cases of resolved CSC, it is difficult to judge if EZ is discontinuous or damaged just by the impression of the examiner alone. Actually, in the current study, out of 25 eyes, rEZc of 8 eyes ranged from 0.40 to 0.69, which means that EZ was moderately damaged. Our results suggested that quantitative assessment of EZ by applying the binarization technique enabled the identification of EZ in all eyes with a sufficient reliability as suggested by the high reproducibility and the tight structure-function relationship.

Therefore, the integrity of EZ should be quantified for precise investigation of the visual function. In this study, we have proposed a novel method for the quantification of EZ in the macula, using binarized images. The quantified index was determined to have statistically significant correlation with VA and MS. Hariris’ quantification method of preserved EZ area with en-face OCT images for RP showed an extremely high ICC of 0.996 (95% CI, 0.995–0.997; p < 0.001)^26^. In this study, we quantified the integrity of EZ using spectral domain OCT (SD-OCT); however, the ICC values were similarly high (0.94: rEZc and 0.98: rEZave).

In this study, the optimal model suggested that rEZc and VA are associated, suggesting that VA reflects retinal function in the fovea. Consistent with the relationship between rEZc and VA, the integrity of EZ within 1mm of the macula was correlated with LogMAR VA more strongly than that within 3 mm of the macula. On the contrary, rEZave was related to MS which indicates that MS reflects retinal function in a wider area around the fovea. We have recently reported that VA was significantly related to the RS within 2 degrees of the fovea measured by the MP-3 microperimeter, but not the RS obtained from wider retinal area in chronic CSC patients^30^. Our current results are in accordance of the previous report. In resolved CSC, even after VA recovers to normal, some patients continue to experience symptoms, such as metamorphopsia and loss of contrast sensitivity^31^. Even after the recovery of VA, RS measured using MP-1 microperimeter was reported as decreased in resolved CSC compared with that of the control eyes^32^. With the automatic tracking system for the retina, the MP-3 microperimeter can project target lights directly to the retina, rather than to the screen, and as a result, they stimulate an intended identical retinal location, even when there is an eye movement. Recently, we have demonstrated the merit of such an approach in eyes with glaucoma; the measured RS had better structure-function relationship than a perimetry without the automatic retinal tracking system^33^. Therefore, in this study, we used the MP-3 microperimeter and as a result, the RS measured using the MP-3 microperimeter was significantly correlated to the integrity of EZ. Many of the other macular diseases, such as cone dystrophy, age-related macular degeneration, and Stargardt disease, are associated with greater decrease in VA and/or central visual sensitivity loss. Consequently, the fixation status becomes poor during VF measurement and also VF measurement can be inaccurate, without the automatic retinal tracking system^34^. The VF measured using the MP-3 microperimeter can be considered reliable even with poor fixation, and would be of further interest to investigate the relationship between the integrity of EZ and RS measured with the MP-3 microperimeter in these diseases.

Our result of the multivariate regression analysis indicated that age was negatively correlated with RS, like the previous reports. Ismail *et al*. divided 50 healthy subjects into four age groups and investigated the influence of age on macular sensitivity. They demonstrated a linear decline in RS with increasing age^35^. Gella and associates prospectively investigated the RS in the central 20 degrees of the macula of 144 healthy volunteers aged from 25 to 69 years, and the linear regression analysis revealed a 0.04 dB/year decline in MS^36^. Even in healthy eyes, the decrease in RS is one of the natural age-related changes. There are previous reports that have suggested a reduction of subfoveal choroidal thickness after spontaneous resolution in acute CSC^43^. In this study, on the contrary, CChT was not related to LogMAR VA and MS.

This study has several limitations. Firstly, the study design was retrospective, and the number of enrolled eyes was less. Secondly, the EZ lines on OCT images were manually identified by examiners. If the image recognition technology, such as deep learning, is exploited to automatically identify the EZ, our method can be more accurate and established. Although this approach usually requires a much large number of samples, future studies should be conducted. Additionally, in the present study, residual EZ was investigated only in CSC, and hence the residual EZ was easily identified, because of the relatively maintained retinal structure. Further studies are needed to examine the usefulness of the current quantification of residual EZ in other retinal diseases, such as cone dystrophy, age-related macular degeneration, and Stargardt disease, in which the outer retinal structure is severely deteriorated.

In conclusion, the quantification of residual EZ had a high interrater reliability and the obtained value was closely related to visual function in patients with resolved CSC.

## Methods

This study was a retrospective observational case series and was conducted at The University of Tokyo, School of Medicine, Tokyo, Japan. Institutional Review Board (IRB) approval was obtained from the University of Tokyo Hospital. All data were fully anonymized before we accessed them. Written informed consent was not required by the IRB but participants who did not grant authorization to use their medical records for the research were excluded from analyses. This study was performed according to the tenets of the Declaration of Helsinki.

We examined, consecutively, 25 eyes of 23 patients (15 males and 8 females) with resolved CSC at the retina clinic at The University of Tokyo Hospital. All patients underwent comprehensive ophthalmic examination including refractive error evaluation, best-corrected visual acuity (BCVA), intraocular pressure, anterior segment examination, and fundus biomicroscopy with pupil dilation. OCT, fluorescein angiography, and indocyanine green angiography were also performed to diagnose patients as having CSC. Eyes with other macular diseases such as choroidal neovascularization, AMD, and intraocular inflammation were excluded.

**Figure 4:**
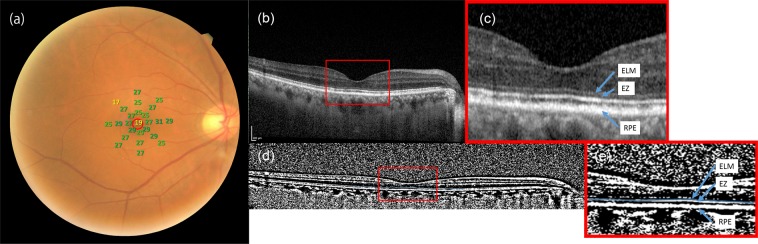
Fundus photograph with visual sensitivities and OCT images in a representative case. Right eye of a 61-year-old man with resolved CSC. The logMAR VA was −0.0792, MS was 26.3. rEZc, rEZave and rEZ1mm were 0.906, 0.971 and 0.829, respectively. (**a**) Retinal sensitivity measurements with the MP-3 microperimeter superimposed onto a fundus photograph. Twenty-five measurement points are located within central 12° of the macula. (**b**,**c**) An OCT image obtained with Spectralis OCT. EZ is located between ELM and RPE. (**d**,**e**) A binarized OCT image. OCT images were binarized using the Niblack method with ImageJ software. Blue arrows indicate the location of ELM, EZ and RPE. The EZ within 3 mm across the fovea was traced by hand (blue line). OCT: optical coherence tomography, CSC: central serous chorioretinopathy, LogMAR: logarithm of the minimum angle of resolution, VA: visual acuity, MS: mean sensitivity, rEZc: the rEZ index of the central horizontal line scan across the fovea, rEZave: the average of rEZ indices of 13 line scans within 3 × 3-mm area, rEZ1mm: the average of rEZ indices of 5 line scans within 1 × 1-mm area of the fovea, EZ: ellipsoid zone, ELM: external limiting membrane, RPE: retinal pigment epithelium.

The BCVA was measured with a Landolt chart and converted LogMAR VA for the analyses. Retinal sensitivity was assessed using the MP-3 microperimeter (Nidek Co. ltd., Japan). The automatic tracking system of the MP-3 microperimeter for fundus movements allows accurate measurement of RS in various ocular diseases^33,37^. The MP-3 measurement was performed with 4–2 full-threshold staircase strategy using the standard Goldmann III stimulus size, similar to previous studies^33,38^. Twenty-five measurement points were located within central 12 degrees of the macular area (Fig. 4). The MP3 microperimetry measurement was conducted centered at fovea. In addition, it was very carefully monitored that visual field grid was well centered at fovea throughout the MP3 microperimetry measurements in all eyes. Only reliable VFs with the following criteria were used for analyses: a fixation loss rate < 20% and a false-positive rate < 15% following our previous report^30^. We calculated the average of the thresholds of 25 measurement points (mean sensitivity: MS).

Simultaneously we obtained SD-OCT images using the Spectralis OCT (Heidelberg Engineering, Germany). For each eye, 13 line scans within 3 × 3-mm area of the macula were obtained. First, the original OCT image was viewed on the ImageJ software (U.S. National Institute of Health, MD). The images were then binarized using the Niblack method. Followingly, EZ within 3 mm square area of the fovea was manually traced by two graders (AF and YA) on the binarized image, referring to the original OCT image (Fig. 4). Once we traced EZ, the rate of white area on the traced line was automatically calculated, which we regarded as the integrity of residual EZ (rEZ index). External limiting membrane (ELM) and RPE were identifiable even in cases with continuous disruption of EZ. In those cases, we estimated where EZ should have existed on OCT images from the location of ELM and RPE. For each eye, we calculated three indices for residual EZ, which are rEZc, rEZave and rEZ1mm. rEZc stands for the white index of the single horizontal line scan on the fovea. rEZave indicates the average of white indices of 13 line scans within 3 × 3-mm area of the macula. rEZ1mm stands for the average of white indices of 5 line scans within 1mm area of the fovea. Using the enhanced depth imaging mode, we measured CRT, MV, and CChT.

To assess the accuracy (interrater agreement) of our current method, we calculated the ICC of rEZ indices. After evaluating reproducibility, the values obtained by the two examiners were averaged for the subsequent statistical analyses.

We evaluated the relationship between LogMAR VA and five variables of age, rEZc, rEZave, CRT, and CChT, using the linear mixed model whereby eyes were nested within a patient, since two eyes of a patient were included in the current study. We also investigated the relationship between MS and age, rEZc, rEZave, MV, and CChT. Additionally, using the corrected AICc model selection from all of 2^5^ models, we investigated which of rEZc and rEZave was responsible for VA and MS, respectively. The AIC is the common statistical measure with which optimal variables can be determined without having an over-fit problem, unlike the coefficient of determination^39^. AICc gives an accurate estimation even when the sample size is small^40^. It is recommended to use model selection methods, instead of multivariate regression, to improve the model fit by removing redundant variables, because the degrees of freedom decrease as the number of variables increases^41,42^. The log-likelihood values of a paired models were compared using the ANOVA. Similar analysis was conducted for MS, instead of LogMAR VA.

All statistical analyses were performed using the R statistical programming language (R version 3.4.3; The Foundation for Statistical Computing, Austria).

## Data Availability

The datasets generated and analysed during the current study are available from the corresponding author on reasonable request.

## References

[1] Daruich A (2015). Central serous chorioretinopathy: Recent findings and new physiopathology hypothesis. Prog Retin Eye Res..

[2] Ojima Y (2007). Three-dimensional imaging of the foveal photoreceptor layer in central serous chorioretinopathy using high-speed optical coherence tomography. Ophthalmology..

[3] Vogel RN (2017). High-resolution imaging of intraretinal structures in active and resolved central serous chorioretinopathy. Invest Ophthalmol Vis Sci..

[4] Ahn SE (2013). Three-dimensional configuration of subretinal fluid in central serous chorioretinopathy. Invest Ophthalmol Vis Sci..

[5] Chung HW (2014). Retinal sensitivity assessed by microperimetry and corresponding retinal structure and thickness in resolved central serous chorioretinopathy. Eye (Lond)..

[6] Wang M, Sander B, la Cour M, Larsen M (2005). Clinical characteristics of subretinal deposits in central serous chorioretinopathy. Acta Ophthalmol Scand..

[7] Baran NV, Gurlu VP, Esgin H (2005). Long-term macular function in eyes with central serous chorioretinopathy. Clin Exp Ophthalmol..

[8] Wu Z, Ayton LN, Guymer RH, Luu CD (2013). Second reflective band intensity in age-related macular degeneration. Ophthalmology..

[9] Tao LW, Wu Z, Guymer RH, Luu CD (2016). Ellipsoid zone on optical coherence tomography: a review. Clin Exp Ophthalmol..

[10] Ojima Y (2010). Restoration of outer segments of foveal photoreceptors after resolution of central serous chorioretinopathy. Jpn J Ophthalmol..

[11] Schocket LS (2006). Ultrahigh-resolution optical coherence tomography in patients with decreased visual acuity after retinal detachment repair. Ophthalmology..

[12] Nguyen MH (2007). Microstructural abnormalities in MEWDS demonstrated by ultrahigh resolution optical coherence tomography. Retina..

[13] Li D, Kishi S (2007). Loss of photoreceptor outer segment in acute zonal occult outer retinopathy. Arch Ophthalmol..

[14] Ota M (2008). Foveal photoreceptor layer in eyes with persistent cystoid macular edema associated with branch retinal vein occlusion. American journal of ophthalmology..

[15] Aizawa S (2009). Correlation between visual function and photoreceptor inner/outer segment junction in patients with retinitis pigmentosa. Eye (Lond)..

[16] Mitamura Y, Aizawa S, Baba T, Hagiwara A, Yamamoto S (2009). Correlation between retinal sensitivity and photoreceptor inner/outer segment junction in patients with retinitis pigmentosa. Br J Ophthalmol..

[17] Baba T (2008). Correlation of visual recovery and presence of photoreceptor inner/outer segment junction in optical coherence images after successful macular hole repair. Retina..

[18] Spaide RF, Koizumi H, Freund KB (2008). Photoreceptor outer segment abnormalities as a cause of blind spot enlargement in acute zonal occult outer retinopathy-complex diseases. Am J Ophthalmol..

[19] Hood DC (2011). The inner segment/outer segment border seen on optical coherence tomography is less intense in patients with diminished cone function. Invest Ophthalmol Vis Sci..

[20] Sundaram, V. *et al*. Retinal structure and function in achromatopsia: implications for gene therapy. *Ophthalmology*. 121–245 (2014). 10.1016/j.ophtha.2013.08.017 PMC3895408 24148654

[21] Matsumoto H, Sato T, Kishi S (2009). Outer nuclear layer thickness at the fovea determines visual outcomes in resolved central serous chorioretinopathy. Am J Ophthalmol..

[22] Hasegawa T, Okamoto M, Masuda N, Ueda T, Ogata N (2015). Relationship between foveal microstructures and visual outcomes in eyes with resolved central serous chorioretinopathy. Graefes Arch Clin Exp Ophthalmol..

[23] Hood DC (2011). Method for deriving visual field boundaries from OCT scans of patients with retinitis pigmentosa. Biomed Opt Express..

[24] Birch DG (2013). Spectral-domain optical coherence tomography measures of outer segment layer progression in patients with X-linked retinitis pigmentosa. JAMA Ophthalmol..

[25] Smith TB (2016). Structure-function modeling of optical coherence tomography and standard automated perimetry in the retina of patients with autosomal dominant retinitis pigmentosa. PLoS One..

[26] Hariri AH (2016). Quantification of ellipsoid zone changes in retinitis pigmentosa using en face spectral domain-optical coherence tomography. JAMA Ophthalmol..

[27] Hartong DT, Berson EL, Dryja TP (2006). Retinitis pigmentosa. Lancet..

[28] Nakamura T, Ueda-Consolvo T, Oiwake T, Hayashi A (2016). Correlation between outer retinal layer thickness and cone density in patients with resolved central serous chorioretinopathy. Graefes Arch Clin Exp Ophthalmol..

[29] Ojima Y (2008). Retinal sensitivity measured with the micro perimeter 1 after resolution of central serous chorioretinopathy. Am J Ophthalmol..

[30] Sugiura A (2017). The association between visual function and retinal structure in chronic central serous chorioretinopathy. Sci Rep..

[31] Nicholson B, Noble J, Forooghian F, Meyerle C (2013). Central serous chorioretinopathy: update on pathophysiology and treatment. Surv Ophthalmol..

[32] Ozdemir H, Karacorlu SA, Senturk F, Karacorlu M, Uysal O (2008). Assessment of macular function by microperimetry in unilateral resolved central serous chorioretinopathy. Eye (Lond)..

[33] Matsuura M (2018). Evaluating the usefulness of MP-3 microperimetry in glaucoma patients. Am J Ophthalmol..

[34] Matsuura M, Hirasawa K, Murata H, Asaoka R (2015). The relationship between visual acuity and the reproducibility of visual field measurements in glaucoma patients. Invest Ophthalmol Vis Sci..

[35] Ismail SA, Sharanjeet K, Mutalib HA, Ngah NF (2015). Macular retinal sensitivity using MP-1 in healthy Malaysian subjects of different ages. J Optom..

[36] Gella L, Nittala MG, Raman R (2014). Retinal sensitivity in healthy Indians using microperimeter. Indian J Ophthalmol..

[37] Asahina Y (2017). The structure-function relationship measured with optical coherence tomography and a microperimeter with auto-tracking: the MP-3, in patients with retinitis pigmentosa. Sci Rep..

[38] Igarashi N (2016). Assessing visual fields in patients with retinitis pigmentosa using a novel microperimeter with eye tracking: the MP-3. PLoS One..

[39] Nakagawa S, Schielzeth H (2013). A general and simple method for obtaining R2 from generalized linear mixed-effects models. Methods Ecol Evol..

[40] Burnham KP, & DR, Multimodel A (2004). inference: understanding: AIC and BIC in model selection. Sociol Methods Res..

[41] Tibshirani RJ, Taylor J (2012). Degrees of freedom in lasso problems. Ann Statist..

[42] Mallows C (1973). Some comments on Cp. Technometrics..

